# Comparative Effects of Intraduodenal Glucose and Fat Infusion on Blood Pressure and Heart Rate in Type 2 Diabetes

**DOI:** 10.3389/fnut.2020.582314

**Published:** 2020-11-09

**Authors:** Cong Xie, Xuyi Wang, Karen L. Jones, Michael Horowitz, Zilin Sun, Tanya J. Little, Christopher K. Rayner, Tongzhi Wu

**Affiliations:** ^1^Adelaide Medical School and Centre of Research Excellence (CRE) in Translating Nutritional Science to Good Health, The University of Adelaide, Adelaide, SA, Australia; ^2^Department of Endocrinology, Zhongda Hospital, Institute of Diabetes, School of Medicine, Southeast University, Nanjing, China; ^3^Endocrine and Metabolic Unit, Royal Adelaide Hospital, Adelaide, SA, Australia; ^4^Department of Gastroenterology and Hepatology, Royal Adelaide Hospital, Adelaide, SA, Australia

**Keywords:** postprandial blood pressure, heart rate, glucose, fat, enteral nutrients, type 2 diabetes

## Abstract

The interaction of nutrients with the small intestine modulates postprandial cardiovascular function. Rapid small intestinal nutrient delivery may reduce blood pressure markedly, particularly in patients with type 2 diabetes (T2DM). Postprandial hypotension occurs in ~30% of patients with longstanding T2DM, but there is little information about the cardiovascular effects of different macronutrients. We compared the blood pressure and heart rate responses to standardized intraduodenal glucose and fat infusions in T2DM. Two parallel groups, including 26 T2DM patients who received intraduodenal glucose infusion and 14 T2DM patients who received intraduodenal fat, both at 2 kcal/min over 120 min, were compared retrospectively. Blood pressure and heart rate were measured at regular intervals. Systolic blood pressure was stable initially and increased slightly thereafter in both groups, without any difference between them. Diastolic blood pressure decreased in response to intraduodenal glucose, but remained unchanged in response to lipid, with a significant difference between the two infusions (*P* = 0.04). Heart rate increased during both intraduodenal glucose and lipid infusions (*P* < 0.001 each), and the increment was greater in response to intraduodenal fat than glucose (*P* = 0.004). In patients with T2DM, intraduodenal fat induced a greater increase in heart rate, associated with a diminished reduction in blood pressure, when compared with isocaloric glucose. The macronutrient composition of meals may be an important consideration in T2DM patients with symptomatic postprandial hypotension.

## Introduction

Exposure of the small intestine to ingested nutrients is associated with a substantial increase in splanchnic blood flow and a decrease in peripheral blood volume, which may lead to a reduction in blood pressure and, in some cases, postprandial hypotension (a fall in systolic blood pressure of ≥20 mmHg within 2 h of a meal) (1). The latter occurs frequently in patients with type 2 diabetes (T2DM), and predisposes to syncope, fall and stroke (1). The rate of gastric emptying (or small intestinal nutrient delivery) is a key determinant of the cardiovascular responses to meals/nutrients in both health and T2DM (1, 2), such that pharmacological or dietary interventions that slow gastric emptying diminish the hypotensive response to meals (3). However, it remains controversial as to whether the gut-provoked changes in cardiovascular function are also dependent on the type of macronutrient. For example, in healthy older individuals, oral carbohydrate, but not fat or protein, induced a hypotensive response (4), whereas intraduodenal infusion of glucose, fat and protein (~3 kcal/min) was reported to reduce blood pressure and increase heart rate comparably, although the timing of these changes differed slightly between them (5). While the use of intraduodenal, as opposed to oral, nutrient administration ensures that the small intestinal exposure to each nutrient is standardized, the rate of intraduodenal infusion employed in the previous studies has approximated the upper-end of the physiological range of gastric emptying (i.e., 1–4 kcal/min) (6), which may limit the capacity to discriminate the effects of different nutrients. Moreover, the comparative effects of different nutrients on blood pressure and heart rate have not been investigated in patients with T2DM.

In the current study, we compared the blood pressure and heart rate responses to intraduodenal glucose and fat infusions at a rate of 2 kcal/min in patients with T2DM.

## Methods and Materials

### Participants

Forty participants with type 2 diabetes managed by diet or metformin monotherapy (500–2,000 mg/day, stable for > 3 months) were recruited from the community by advertisement for studies evaluating nutritional and/or pharmacological therapies for diabetes in our center. Twenty-six participants received an intraduodenal glucose infusion (7, 8) and 14 participants received an intraduodenal lipid infusion (9) following identical procedures after providing written informed consent (Table 1). We have previously reported the outcomes relating to blood glucose and gut hormones. We now report the data on blood pressure and heart rate collected from these studies. None had impaired liver or renal function, significant gastrointestinal disease or prior gastrointestinal surgery, or diabetic microvascular complications, including autonomic dysfunction as assessed using standardized cardiovascular reflex tests. All antihypertensive medications were withheld for 24 h prior to the study. The protocols were approved by the Royal Adelaide Hospital Human Research Ethics Committee.

**Table 1:** Subject characteristics.

	**Glucose**	**Lipid**	***P*-value**
Gender (male/female)	21/5	9/5	0.3
Age (years)	65 ± 2.3	68 ± 2.4	0.1
BMI (kg/m^2^)	30.3 ± 1.5	30.7 ± 1.3	0.8
HbA1c (%)	6.4 ± 0.1	6.7 ± 0.2	0.1
HbA1c (mmol/mol)	46.7 ± 1.1	49.9 ± 2.1	0.1
Duration of known diabetes (years)	4.5 ± 1	6.5 ± 1.5	0.2
Fasting blood glucose (mmol/l)	7.7 ± 0.3	7.9 ± 0.7	0.8
Use of hypotensive medication	7/26	3/14	0.2
Basal systolic blood pressure (mmHg)	131.4 ± 3	136.1 ± 5.6	0.4
Basal diastolic blood pressure (mmHg)	75 ± 1.6	72.3 ± 2.4	0.3
Basal heart rate (beats/min)	58.1 ± 1.7	61.6 ± 2.9	0.3

*Data are mean values ± SEM*.

### Protocol

Blood pressure and heart rate were evaluated on a single study visit. All participants refrained from strenuous physical activity for 24 h before the study. After a standardized evening meal (McCain beef lasagne, Victoria, Australia) at ~1,900 h and an overnight fast, participants attended the laboratory at 0800 h. A multi-lumen silicone catheter (Dentsleeve International Ltd, Mui Scientific, Ontario, Canada) was inserted transnasally and allowed to pass into the duodenum by peristalsis, as described (5). An infusion port opened 12 cm beyond the pylorus. Commencing at *t* = 0 min, either glucose or lipid (20% Intralipid, consisting of long-chain triglycerides; Fresenius Kabi AB, Sweden) was infused at 2 kcal/min over 120 min (*t* = 0–120 min) while the participant was lying supine. Systolic and diastolic blood pressure and heart rate were measured regularly (glucose infusion group: every 5 min; lipid infusion group: every 3 min) from 60 min before starting infusion using an automatic sphygmomanometer (GE Healthcare, Milwaukee, WI, United States). Mean blood pressure and heart rate every 15 min were reported. Data on heart rate during intraduodenal lipid infusion were only available for nine patients only due to technique issues. Baseline blood pressure and heart rate were calculated as an average of the measurements obtained 15 min prior to intraduodenal infusion.

### Statistical Analysis

Demographic data in the two groups were compared using unpaired Student's *t*-tests after confirming their normality of distribution, with the exception that the proportions of gender and use of antihypertensive medication were compared using Fisher's exact test. Blood pressure and heart rate responses to intraduodenal infusions were evaluated using two-way repeated measures ANOVA, with treatment and time as factors. *Post hoc* comparisons, adjusted for multiple comparisons by Bonferroni's correction, were performed if ANOVAs revealed significant interactions. Analyses were performed using Prism 8.0 (La Jolla, CA, United States). *P*-value less than 0.05 was considered statistically significant. Data are expressed as means ± SEM.

## Results

**Figure 1:**
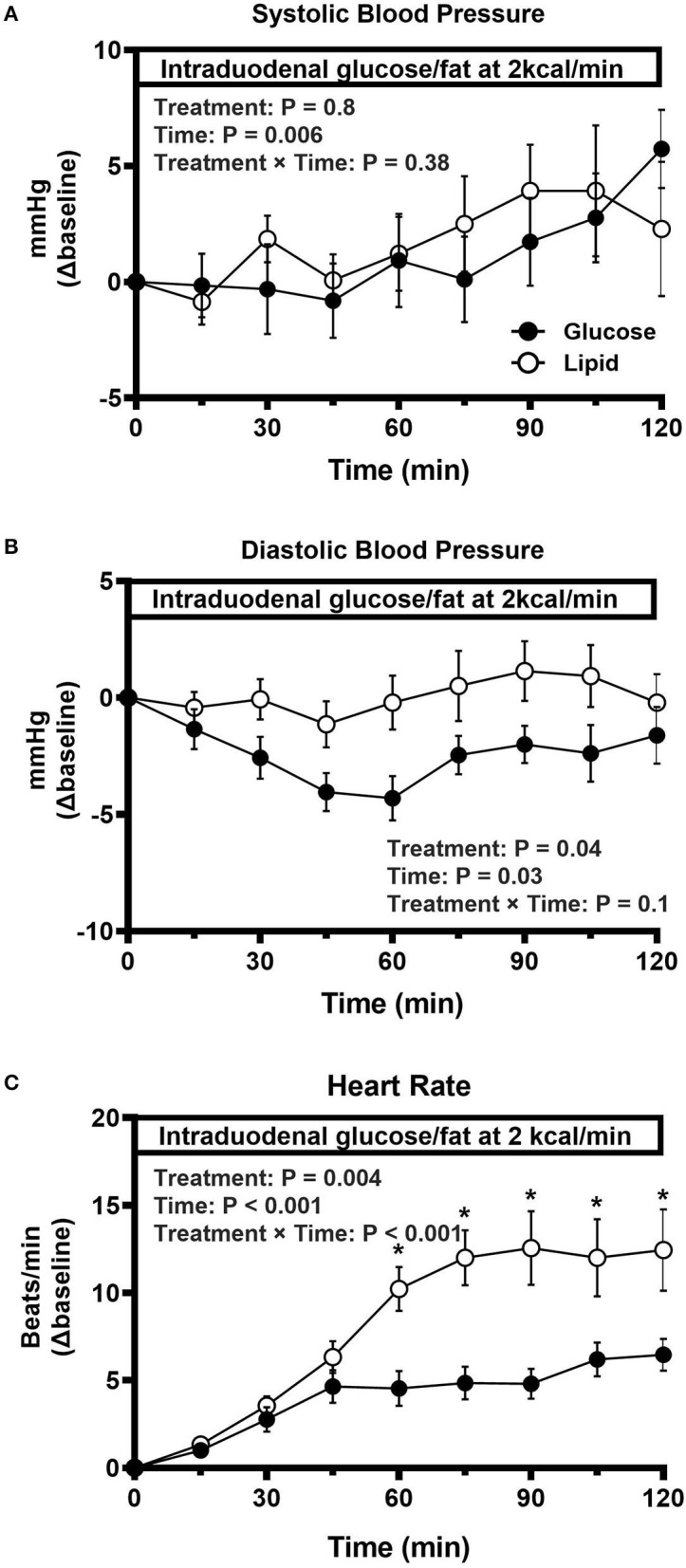
Change in systolic blood pressure **(A)**, diastolic blood pressure **(B)**, and heart rate **(C)** in response to intraduodenal glucose or fat infusion at 2 kcal/min (*t* = 0–120 min) in type 2 diabetes. Repeated-measures ANOVA was used to determine statistical difference. Results of ANOVA are reported as *P*-values for differences over treatment, difference by time and differences due to treatment by time interaction. *Post hoc* comparisons were adjusted by Bonferroni's correction. If ANOVA results were significant. ^*^P < 0.05 for each. Data are mean values ± SEM.

All participants tolerated the study well and none exhibited a fall in systolic blood pressure ≥20 mmHg during the study. Baseline characteristics, including age, sex, BMI, HbA1c, fasting blood glucose, use of antihypertensive medication, fasting systolic and diastolic blood pressure and heart rate, were well matched between the two groups (Table 1). During intraduodenal infusion, systolic blood pressure remained relatively stable for the first 60 min, and increased slightly thereafter in both groups (time effect: *P* < 0.001 during glucose, and *P* = 0.14 during lipid, infusion), without significant difference between them (Figure 1A). Diastolic blood pressure decreased by ~5 mmHg within the first 60 min followed by a slow recovery during glucose infusion (time effect: *P* < 0.001), but remained unchanged during lipid infusion (time effect: *P* = 0.61), such that the reduction in diastolic blood pressure was greater in response to intraduodenal glucose than lipid (treatment effect: *P* = 0.04) (Figure 1B). Heart rate increased progressively within the first 60 min and plateaued thereafter during both infusions [time effect: *P* < 0.001 for each). The increment in heart rate was greater in response to intraduodenal lipid than glucose (*P* = 0.004 for treatment effect; *P* < 0.001 for treatment by time interaction, with significant differences between *t* = 60–105 min (*P* < 0.05 for each)] (Figure 1C).

## Discussion

In patients with relatively well-controlled, uncomplicated T2DM, we observed a reduction in diastolic blood pressure in response to intraduodenal glucose infusion at a rate of 2 kcal/min, but not to an isocaloric fat infusion, probably due to its greater effect to increase heart rate. These observations suggest that the macronutrient composition of a meal may have a major impact on postprandial cardiovascular function in T2DM.

In contrast to previous observations made in healthy older participants during intraduodenal infusion of glucose and fat at a higher rate (~3 kcal/min) (5), there were minimal changes in systolic blood pressure in response to intraduodenal glucose and lipid infusion in the present study. This is likely to reflect that the hypotensive response to enteral nutrients is subject to the rate of small intestinal nutrient delivery (2), and that intraduodenal infusion of glucose and lipid at a rate of 2 kcal/min may not be sufficient to induce a marked reduction in systolic blood pressure (2). In addition, we observed a greater increase in heart rate during intraduodenal fat than glucose infusion, which may account for the observed absence of reduction in diastolic blood pressure. The apparent lack of any difference in the blood pressure and heart rate responses to glucose and fat observed previously may reflect near maximal changes in blood pressure and heart rate at the higher rate of nutrient infusion.

The mechanisms underlying the differences in the blood pressure and heart rate responses to intraduodenal glucose and lipid remain to be defined, but are unlikely to be related to peripheral glucose and insulin levels (1). We have shown that intraduodenally administered fat (at 2 kcal/min) is more potent than glucose at stimulating the secretion of the “incretin” hormones glucagon-like peptide-1 (GLP-1) and glucose-dependent insulinotropic polypeptide (GIP) (10), which have the capacity to modulate cardiovascular function in humans. For example, administration of both exogenous GLP-1 and GLP-1 receptor agonists has been shown to increase heart rate and attenuate the hypotensive response to an oral glucose or intraduodenal glucose infusion in both healthy subjects and patients with T2DM (3, 11–13). Conversely, blockade of endogenous GLP-1 signaling by the GLP-1 receptor antagonist, exendin (9–39), diminishes the heart rate increase in response to intrajejunal glucose infusion in patients with T2DM (14). While the role of endogenous GIP signaling in the regulation of blood pressure and heart rate has not been investigated in detail, exogenous GIP has been reported to decrease blood pressure and increase heart rate in individuals with impaired glucose tolerance (15). In addition, differences in the digestive requirements between glucose and fat may be of relevance. In support of this concept, inhibition of lipid digestion by orlistat was shown to attenuate the heart rate response to intraduodenal fat infusion (at 3 kcal/min) in healthy older humans, although blood pressure was not affected (16).

In interpreting our findings, several limitations should be noted. First, our model of infusing either glucose or fat directly into the duodenum is, by definition, non-physiological, and does not allow us to evaluate the contribution of gastric factors to the regulation of postprandial blood pressure and heart rate. However, it is ideal to differentiate the blood pressure and heart rate changes mediated by the small intestine in response to glucose and fat, by standardizing the delivery of nutrients into the small intestine. Bypassing the stomach eliminated the potential influence of gastric emptying on the postprandial blood pressure and heart rate responses (17). Moreover, it circumvented the impact of differing degrees of gastric distension arising from variations in the rate of gastric emptying between individuals (18, 19). Second, we did not measure cardiac hemodynamics, cardiac output and muscle sympathetic activity, which would have provided a more comprehensive characterization of postprandial cardiovascular function. Third, the sample sizes were relatively small; however, the findings appeared clear-cut. Finally, the T2DM patients were relatively well-controlled and had no evidence of cardiovascular autonomic dysfunction, which may in part have accounted for the fact that the falls in blood pressure were modest. Evaluation of patients with more advanced T2DM, particularly in those prone to postprandial hypotension, would be of interest.

In summary, when administered at identical energy loads, fat increases heart rate more than glucose, associated with a diminished reduction in blood pressure, in patients with relatively well-controlled, uncomplicated T2DM. These observations highlight the distinct impact of different nutrients on postprandial cardiovascular function and suggest that the macronutrient composition of meals may be an important consideration in T2DM patients with symptomatic postprandial hypotension.

## Data Availability Statement

The datasets presented in this article are not readily available because the dataset cannot be publicly due to the restrictions from the ethics committee. Requests to access the datasets should be directed to tongzhi.wu@adelaide.edu.au.

## Ethics Statement

The studies involving human participants were reviewed and approved by Royal Adelaide Hospital Human Research Ethics Committee. The patients/participants provided their written informed consent to participate in this study.

## Author Contributions

CX and XW were involved in conception, design and coordination of the study, subject recruitment, data collection and interpretation, statistical analysis, and writing of the manuscript. ZS, KJ, MH, TL, CR, and TW were all involved in conception, design, and data interpretation. All authors critically reviewed the manuscript and have read and approved the publication of this final version of the manuscript. All authors contributed to the article and approved the submitted version.

## Conflict of Interest

KJ has received research funding from Sanofi and AstraZeneca and drug supplies from Merck Sharp & Dohme. MH has participated in the advisory boards and/or symposia for Novo Nordisk, Sanofi, Novartis, Eli Lilly, Merck Sharp & Dohme, Boehringer Ingelheim, and AstraZeneca and has received honoraria for this activity. CR has received research funding from AstraZeneca, Merck Sharp & Dohme, Eli Lilly, Novartis, and Sanofi. TW has received travel support from Novartis and research funding from Novartis and AstraZeneca. TL has received research funding from Novartis. The remaining authors declare that the research was conducted in the absence of any commercial or financial relationships that could be construed as a potential conflict of interest.
